# High rate of in-stent restenosis after coronary intervention in carriers of the mutant mannose-binding lectin allele

**DOI:** 10.1186/s12872-016-0440-y

**Published:** 2017-01-05

**Authors:** Zsolt Bagyura, Loretta Kiss, Balázs Berta, Ágnes Szilágyi, Kristóf Hirschberg, Gábor Széplaki, Árpád Lux, Zsolt Szelid, Pál Soós, Béla Merkely

**Affiliations:** 1 Heart and Vascular Center, Semmelweis University, Varosmajor utca 68, Budapest, H-1122 Hungary; 2 3rd Department of Internal Medicine, Research Laboratory, Semmelweis University, Budapest, Hungary; 3 Department of Cardiology, University Hospital Heidelberg, Im Neuenheimer Feld 410, 69120 Heidelberg, Germany

**Keywords:** In-stent restenosis, MBL genetic variant, Bare metal stent, Gene association, Cardiology

## Abstract

**Background:**

In-stent restenosis occurs in 10–30% of patients following bare metal stent (BMS) implantation and has various risk factors. Mannose-binding lectin (MBL) is known to have effect on the progression of atherosclerosis. Single nucleotide polymorphisms (SNP) of the MBL2 gene intron 1 (codon 52, 54, 57) are known to modulate the bioavailability of the MBL protein. Our aim was to identify the association of these polymorphisms of the MBL gene in the occurrence of in-stent restenosis after coronary artery bare metal stent implantation.

**Methods:**

In a non-randomized prospective study venous blood samples were collected after recoronarography from 225 patients with prior BMS implantation. Patients were assigned to diffuse restenosis group and control group based on the result of the coronarography. MBL genotypes were determined using quantitative real-time PCR. Proportion of different genotypes was compared and adjusted with traditional risk factors using multivariate logistic regression.

**Results:**

Average follow-up time was 1.0 (+ − 1.4) year in the diffuse restenosis group (*N* = 117) and 2.7 (+ − 2.5) years in the control group (*N* = 108). The age, gender distribution and risk status was not different between study groups. Proportion of the MBL variant genotype was 26.8% (29 vs. 79 normal homozygous) in the control group and 39.3% (46 vs. 71 normal homozygous) in the restenosis group (*p* = 0.04). In multivariate analysis the mutant allele was an independent risk factor (OR = 1.96, *p* = 0.03) of in-stent restenosis.

**Conclusions:**

MBL polymorphisms are associated with higher incidence of development of coronary in-stent restenosis. The attenuated protein function in the mutant allelic genotype may represent the underlying mechanism.

## Background

Coronary interventions revolutionised the treatment of acute and stable forms of coronary artery disease (CAD) [1]. However, after balloon angioplasty restenosis of the target segment of the vessel occurs in 40–50% [2]. Coronary stents were designed to lower the rate of early restenosis. The most common complications of stent implantation are stent thrombosis and in-stent restenosis (ISR). While stent thrombosis is known to be associated with thrombocyte function, ISR is associated with endothel proliferation. ISR still occurs in 10–30% of the interventions with deployment of bare metal stents (BMS), therefore it still forms a clinically important problem [3]. With the use of drug eluting stents (DES) or other modern stents designed to lower the ratio of endothel proliferation, restenosis ratio could be further reduced. However, indication of these stents is less wide and the cost of these stents is significantly higher, therefore BMS’s are still widely used. Risk factors for in-stent restenosis could be classified in two groups. Procedural-dependent or local factors are: diameter of vessel, length of lesion or stent, minimal lumen diameter before and after stenting, ostial lesions, stent fracture, total occlusions [4]. The other group consists of patient-dependent or general factors. Risk for restenosis is particularly high among patients with diabetes mellitus, this may be associated with metabolic alterations that promote endothelial dysfunction [5], accelerate intimal hyperplasia [6], and increased platelet aggregability and thrombogenicity. There is evidence that gender itself (female) predisposes to restenosis, and some patients may have a genetically higher risk [7]. Genetic polymorphisms associated with high risk for restenosis include polymorphisms in genes coding for angiotensin-converting enzyme inhibitor [8, 9], glycoprotein receptor IIIa [10] and haptoglobin [11]. On invasive coronarography in-stent restenosis can be classified according to the Mehran’s classification to focal (Mehran I) and diffuse (Mehran II-IV) groups [12]. The former type is determined by local and procedural factors, while the latter shows a significant relationship with general, patient-related factors [13].

### Mannose-binding lectin

Mannose-binding lectin (MBL) is an acute phase protein produced by the liver as part of the innate immune system. It also has a direct opsonisation effect by binding to cell-surface receptors on phagocytes. It is present in the blood serum forming a complex with serine proteases [14, 15]. According to the results of prior studies a low level of MBL is related to the rapid progression of atherosclerosis [16], severe coronary artery disease [18] and/or increased carotid plaque formation and graft occlusion after bypass surgery. Functioning alleles show an association with the serum level of MBL protein.

### Genetic background

MBL protein is encoded from the MBL2 gene on chromosome 10. Single nucleotide polymorphisms (SNP) in exon 1 concerning the promoter region of the MBL2 gene are known to reduce the amount of functional MBL subunits 5- to 10-fold, resulting in lower serum levels of MBL: at codon 52 (arginine to cysteine, allele D), codon 54 (glycine to aspartic acid, allele B,) and codon 57 (glycine to glutamic acid, allele C). These variant alleles are commonly named as O, and the normal allele has been named A [19]. Impaired production of MBL is associated with these three SNP-s [20].

Based on these findings that indicate probable involvement of the MBL system in the pathological processes leading to restenosis, and the impaired MBL production related to these SNP-s our study was performed to analyse the association of MBL2’s polymorphisms and the development of coronary in-stent restenosis after bare metal stent deployment.

## Methods

### Subjects, interventions and follow-up

In our non-randomized prospective study, we investigated patients who had re-coronarography in the Heart and Vascular Center, Semmelweis University between 2009 and 2011 due to symptoms of non-acute cardiac event (stable, instable or effort angina pectoris) or acute coronary syndrome. All the patients received BMS during the first percutaneous coronary intervention (PCI), we excluded patients from this study who received DES. All patients received standard therapy according to the actual guidelines. ISR were evaluated by experienced clinicians according to Mehran’s classification.

The restenosis group included patients received BMS during the first PCI and had significant diffuse ISR (Mehran II-IV) at recoronarography. A total number of 117 patients (83 men (70.9%)) were included.

Control group (*n* = 108) formed by patients who received BMS during the first PCI and had recoronarography because of novel complaints, but developed no or only focal restenosis (Mehran I) in the target bare metal stent.

### Biological samples and genotyping

Total genomic deoxyribonucleic acid (DNA) was extracted from EDTA-anticoagulated blood samples collected after the second coronarography using the method of Miller et al. Determination of the alleles of the MBL2 gene at codons 52 (D - rs5030737), 54 (B - rs1800450), and 57 (C - rs1800451) were performed by polymerase chain reaction using sequence-specific priming. The common designation for these variant alleles is O, whereas the normal allele has been named A [19].

### Statistical analyses

Data were collected in Microsoft Excel 2003 and were analysed with SPSS 13.0 for Windows (SPSS, Chicago, USA) software. Categorical variables were reported as absolute numbers and percentages, and continuous variables as medians and interquartile ranges. We used the t-test to compare continuous variables between groups, whereas for continuous nonparametric variables we used the Mann–Whitney U-test. Categorical values were compared by using the chi-square test. Multivariate logistic regression has been performed with adjustment for generally known risk factors and MBL variant genotype (A/O + O/O) with in-stent restenosis as a dependent variable, independent variables were entered into the equation simultaneously. The genotype frequency was tested for deviation from Hardy–Weinberg equilibrium by using Pearson’s chi-square test. All analyses were performed two-tailed, and *p* < 0.05 was considered as significant.

## Results

### Patient characteristics

The average follow-up time was 2.7+/−2.5 years in the control group and 1.0+/−1.4 years in the restenosis group (*p* < 0.0001). According to this diffuse restenosis occurred and caused symptoms earlier while controls had a longer asymptomatic period before re-coronarography was performed. Clinical baseline characteristics of the 225 patients are described in Table 1, dividing study participants in two groups. The mean age (control: 66.4 (+ − 10.8) years vs. restenosis: 65.7 + −9.9 years; *p* = 0.632) and distribution of genders (control: 76.9% male vs. restenosis 70.9% male; *p* = 0.314) did not differ significantly in the two groups.

**Table 1:** Patient characteristics

Characteristics	Control group (*n* = 108)	Restenosis group (*n* = 117)	*p*-value
Age (years)	66.4 (+−10.8)	65.7 (+−9.9)	0.632
Gender, male (%)	83 (76.9%)	83 (70.9%)	0.314
Anti-hypertensive therapy (%)	104 (96.3%)	108 (92.23%)	0.200
Lipid-lowering therapy (%)	98 (90.7%)	107 (91.5%)	0.851
Diabetes mellitus (%)	39 (36.1%)	39 (33.3%)	0.662
Obesity - BMI > 25 (%)	76 (70.4%)	91 (78.4%)	0.165
Smoking	32 (47.1%)	36 (52.9%)	0.927
Multi-vessel disease (%)	10 (9.4%)	19 (16.2%)	0.131
Acute coronary disease (%)	61 (57%)	67 (57.3%)	0.969
Follow up time (years)	2.73 (+−2.54)	1.03 (+−1.38)	<0.001
Total number of stents	1.37 (+−0.54)	1.69 (1.04)	0.005

The presence of risk factors such as hypertension, diabetes, hyperlipidaemia, obesity, multivessel disease did not differ significantly in the groups. About one-third of patients had diabetes (78 patients, 34.7%), mostly type 2. Most patients had hypertension and hyperlipidaemia, only 6% were not treated for hypertension and 9% were not on lipid-lowering therapy and had normal lipid levels. Obesity (body mass index (BMI) >25) was present in 74% of patients. Multiple vascular disease was detected in 49 patients (24.1%) at the time of the recoronarography, 23 (11.3%) cases had the anamnesis of stroke or TIA and 35 (17.2%) cases had peripheral artery disease in the anamnesis. 11 patients had renal failure and 6 patients had cardiogen shock.

### Interventions

There was no significant difference in the indication of stent implantation between the two groups: acute coronary disease in 57% of the control group and 57.3% in the restenosis group. Total number of deployed stents was significantly higher in restenosis group 1.37 (+ − 0.54) vs 1.69 (1.04) (*p* = 0.005). There were only a few patients (*n* = 29, 13%) who had multiple branches stented for the first intervention, 10 (9.4%) in the control group and 19 (16.2%) in the restenosis group (*p* = 0.131).

### Genotype distribution and statistical analysis

Allele frequencies were similar between genders (67.7/31.1/1.2 in men and 67.2/35.6/1.7 in women, *p* = 0.778). The genotype distribution did not deviate from Hardy–Weinberg equilibrium (*p* = 0.08). Proportion of the MBL variant genotype (A/O + O/O) was 26.8% (29 vs. 79 normal homozygous) in the control group and 39.3% (46 vs. 71 normal homozygous) in the restenosis group (OR: 1.784, *p* = 0.04) (Table 2).

**Table 2:** Genotype distribution

MBL genotype	Control group *n* = 108 (%)	Diffuse restenosis group *n* = 117 (%)	*p*-value
Normal (A/A carriers)	79 (73.1%)	71 (60.6%)	0.04
Variant (A/0 + 0/0 carriers)	29 (26.8%)	46 (39.3%)	

A/A vs A/0 + 0/0, Khi-square

Univariate analysis revealed that MBL variant genotype (A/O + O/O) (OR: 1.784, *p* = 0.047) and total number of implanted stents (OR: 0.519 , *p* = 0.034) influence the development of in-stent restenosis. We performed multivariate adjustment for known risk factors (gender, age, BMI, hypertension, dyslipidaemia, diabetes mellitus, smoking, acute coronary disease and total number of implanted stents) and MBL variant genotype (A/O + O/O) as covariates. The regression analysis revealed that MBL variant genotype (OR: 1.95, *p* = 0.03), BMI (OR: 1.08, *p* = 0.03) and total number of implanted stents (OR: 1.64, *p* = 0.01) are independently associated with development of in-stent restenosis (Table 3).

**Table 3:** Results of the multivariate logistic regression analyses with adjustment for generally known risk factors and MBL variant genotype (A/O + O/O) with in-stent restenosis as a dependent variable. Hosmer Lemeshow test *p* = 0.477

Risk factor	OR	C.I.		*P*
		Lower	Upper	
Gender (male)	1.235	0.632	2.413	0.54
Age	0.997	0.969	1.027	0.86
Hypertension or anti-hypertensive therapy	0.388	0.092	1.640	0.20
Hyperlipidaemia or lipid-lowering therapy	0.906	0.304	2.697	0.86
BMI	1.085	1.007	1.170	0.03
Smoking	1.207	0.528	2.763	0.65
Diabetes mellitus	0.798	0.431	1.478	0.47
MBL variant genotype	1.956	1.055	3.626	0.03
Total number of stents	1.648	1.126	2.413	0.01
Acute coronary syndrome	1.185	0.653	2.149	0.58

## Discussion

Mannose-binding lectin is an acute phase protein produced by the liver as part of the innate immune system. In our non-randomized prospective study, multivariate analysis revealed that the MBL variant genotype (A/O + O/O) is independently associated with and has a significant effect upon the development of in-stent restenosis. After coronary artery stent implantation, patients are at a higher risk of diffuse in-stent restenosis if they are carrying variant MBL alleles (A/O or O/O) than those homozygous for the normal MBL A/A genotype. The link between polymorphisms and restenosis is that single nucleotide polymorphisms (SNP) concerning the promoter region of the MBL2 gene are known to influence the bioavailability of MBL by resulting in a lower serum concentration [21].

Clinical observations show that low serum levels of MBL can predispose to several infections, especially those involving the respiratory system and may play a role in certain autoimmune disorders [22, 23]. It has been suggested by others that MBL deficiency also plays a role in several non-infectious diseases such as systemic lupus erythematosus [24], type 2 diabetes mellitus [25], cystic fibrosis [26], rheumatoid arthritis [27] and Crohn’s disease [28].

Previously it has been shown that MBL deficiency may be associated with accelerated atherosclerosis [16, 17] and also coronary plaque formation [29, 18]. Its association with the progression of atherosclerosis can be explained in different ways. Several studies have demonstrated the relationship between chronic Chlamydia pneumoniae (intracellular bacteria) infection and coronary artery stenosis [30, 31]. Given that most people are exposed to this infection during their lifetime, it is feasible that the lack of MBL modifies the quality and/or intensity of the immune reaction against Chlamydia [29]. Higher rate of restenosis may be caused by the prolongation of the inflammatory response and the healing process of the endothelium [32]. Furthermore, MBL has an influence on the progression of atherosclerosis irrespective of neointima hyperplasia.

In contrast, Széplaki et al. found that a significant increase in the frequency of MBL variant genotypes was observed in patients not experiencing restenosis compared with the patients with restenosis after carotid endarterectomy [33]. The higher incidence of early restenosis after carotid endarterectomy was associated with elevated C3 level and carrying the wild type of MBL2 allele [34].

## Limitations

We presented a genetic association study in a relatively small sample size, with considerably wide confidence intervals for odds ratios, therefore a spurious association cannot be fully ruled out. Further studies are needed and sample size should be increased to confirm the results of the present study. Plasma MBL levels could be determined for a better understanding how MBL polymorphisms affect restenosis after percutaneous coronary interventions.

## Conclusions

In our study, multivariate analysis revealed that MBL variant genotype (A/O + O/O) is independently associated with and has a significant effect upon the development of in-stent restenosis. According to our results and the results of others, it is likely that the mechanisms leading to restenosis are different in the carotid and coronary arteries. The MBL genetic system may be a promoter or a protector factor of the atherosclerosis depending on the pathophysiological scenario within the vessel wall and that it is a fine-tuned balance that determines whether complement is an advantage or disadvantage in cardiovascular disease settings. In conclusion, our study shows that MBL polymorphisms’ are associated with the development of coronary in-stent restenosis. The attenuated protein function related to the mutant allelic genotype may represent the underlying mechanism.

## Abbreviations

BMI: Body mass index
BMS: Bare metal stent
CAD: Coronary artery disease
CI: Confidence interval
DES: Drug eluting stent
DNA: Deoxyribonucleic acid
ISR: In-stent restenosis
MBL: Mannose-binding lectine
OR: Odds ratio
PCI: Percutaneous coronary intervention
SNP: Single nucleotide polymorphism
